# Intrauterine and early-life malnutrition in rats disrupts the circadian rhythm programming of energy metabolites through adulthood

**DOI:** 10.1371/journal.pone.0299554

**Published:** 2024-03-27

**Authors:** Dulce Jocelyn Bustamante-Valdez, Martin Alejandro Fuentes-Cano, Jesus Salvador Gonzalez-Ruano, Alonso Martinez-Canabal, Rene Cardenas-Vazquez, Pilar Duran

**Affiliations:** Neurofisiología del Desarrollo y Bioseñales, Laboratorio de Biología Animal Experimental, Depto. Biología Celular, Facultad de Ciencias, Universidad Nacional Autónoma de México, Coyoacán, México; Nippon Medical School, JAPAN

## Abstract

Maternal malnutrition plays a crucial role in functional development, resulting in behavioral, cognitive, and metabolic abnormalities and disturbances. “Cafeteria diet” has been linked to obesity, metabolic syndrome, diabetes, and other metabolic disruptions in the mammalian lifespan. However, there are very few reports about the effect of intrauterine and early postnatal malnutrition on the circadian rhythm programming of energy metabolites. In mammals, circadian rhythm central control is fundamental for correct interaction with the environment and physiological regulation. Exposure to malnutrition during development imprints metabolic programming throughout life on the central nervous system and peripheral systems. Lifespan studies exploring the effect of high fat/low protein diet administered during critical periods of development are scarce. The present study explored the effect of intrauterine and perinatal malnutrition induced by a high fat/low protein diet (Cafeteria Diet) on circadian and peripheral oscillators controlling glucose, insulin, and triglycerides in rats at 40 and 90 days of age. We evaluated plasma glucose and triglyceride levels in 6 Zeitgeber times, in addition to an intraperitoneal glucose tolerance test (IpTGT) and homeostasis model assessment of insulin resistance (HOMA-IR) at two time-points over 24h. Our results show that offspring of malnourished dams fed cafeteria diet present alterations in circadian rhythmicity of glucose and triglycerides associated with a change in glucose tolerance and insulin sensibility differentially regulated at the development stage and time of day. Intrauterine and early malnutrition due to a cafeteria diet produces maladaptive responses and programs energetic metabolism at several developmental stages during the lifespan.

## 1. Introduction

Intrauterine and early malnutrition are crucial in critical developmental periods and functional embryonic and fetal development, resulting in behavioral, cognitive, and metabolic abnormalities and disturbances throughout life [1]. Therefore, maternal malnutrition induced by inappropriate nutrient balance during pregnancy and lactation is a risk factor for both the mother and the fetus since it can alter the development programming [2] and the behavior and metabolism of the offspring [3–5]. Evidence suggests that deficient nutrition in animals during early life stages can be a precondition for adult individuals to develop metabolic pathologies, such as obesity, hyperglycemia, altered insulin sensibility, and lipid metabolism [6–11]. When dams are exposed to highly palatable diets during gestation and lactation, as cafeteria diets, offspring can or may be susceptible to developing alterations in food ingestion, weight gain, adiposity, and metabolism, either during development or at later life stages [9, 11, 12].

Circadian clocks impose the phase and regulate circulating levels of energy metabolites. Circadian clocks’ integrity and hierarchical organization regulate metabolism, i.e., circulating glucose concentrations, lipid homeostasis, and maintaining homeostatic levels that can prevent metabolic diseases such as diabetes and fatty liver [13, 14]. Energy metabolism displays a temporal organization that repeats every 24 h, inducing a food intake rhythm. In mammals, circadian rhythmicity is organized hierarchically, with a central master oscillatory clock, the hypothalamic suprachiasmatic nucleus (SCN), which regulates peripheral oscillators or pacemakers, such as those in the pancreas [15] and liver [14, 16]. The energetic metabolism regulation results from coordinated hormone release and metabolic pathways activity [14, 16–18]. The central oscillator regulates the peripheral oscillators upholding a controlled phase relationship that promotes the circadian release of hormones, such as insulin and glucagon, needed to control blood glucose and triglycerides. Also, external factors, such as diet, modulate oscillatory patterns [17, 19–21]; e.g., previous studies showed that high-fat diets dysregulate circadian oscillations of energetic metabolites while increasing caloric demand during resting stages [14, 22, 23].

However, there are few reports about the changes in circadian rhythms of energy metabolites associated with alterations in early developmental stages of the offspring of dams fed an imbalanced diet during pregnancy and lactation. Neither is clear if those changes persist later in life [24]. The present study aimed to analyze in juvenile and young adult rats circadian energy metabolism consequences (as programming) of intrauterine and early postnatal malnutrition induced by a cafeteria diet in dams during pregnancy and lactation. We hypothesize that intrauterine and early-life malnutrition impairs the circadian phase relationship within the peripheral metabolic oscillators, causing rhythmic imbalances in the critical energy metabolites released in juvenile and young adult rats. The results showed substantial modifications in the circadian rhythmicity of glucose, triglycerides, and insulin resistance depending on the analyzed age, sex, and entrainment.

## 2. Materials and methods

### 2.1. Animals and treatment

Experiments were performed in Sprague-Dawley rats born and raised at Facultad de Ciencias, UNAM. Dams were adult nulliparous aged 12–18 weeks and weighing 200–250 g. All subjects were maintained under standard colony conditions in a temperature and light-controlled room (12:12 light-dark cycle, temperature between 22–24°C and humidity 40–50%) and had *ad libitum* access to food and water (Lab Chow 5001, Purina). Lights were on at zeitgeber time (ZT0) (7 am local time) and lights off at ZT12 (7 pm local time). All animals were paired-housed on shavings in polycarbonate cages (45 cm x 20 cm x 25 cm). In order to obtain the experimental offspring, three weeks before mating, 16 adult females were randomly separated into two groups: Control (C) fed with a commercial diet (Purina Lab Chow 5008) and Cafeteria Diet (CD); for mating, one female (when in proestrus confirmed by vaginal smear) and one male were placed together. Pregnancy day one was established when vaginal smears showed the presence of spermatozoids. After birth, littermates were standardized to 10 pups per dam in an equal male/female ratio. After weaning (postnatal day 21), all animals were subjected to Purina Lab Chow 5001, divided by gender and condition, and housed 3 per cage maximum. To minimize homogeneity in the protocol designed groups, one (in some cases two siblings) pup C or CD offspring were randomized, chosen from each litter, and assigned to their related group.

### 2.2. Cafeteria diet

**Fig 1:**
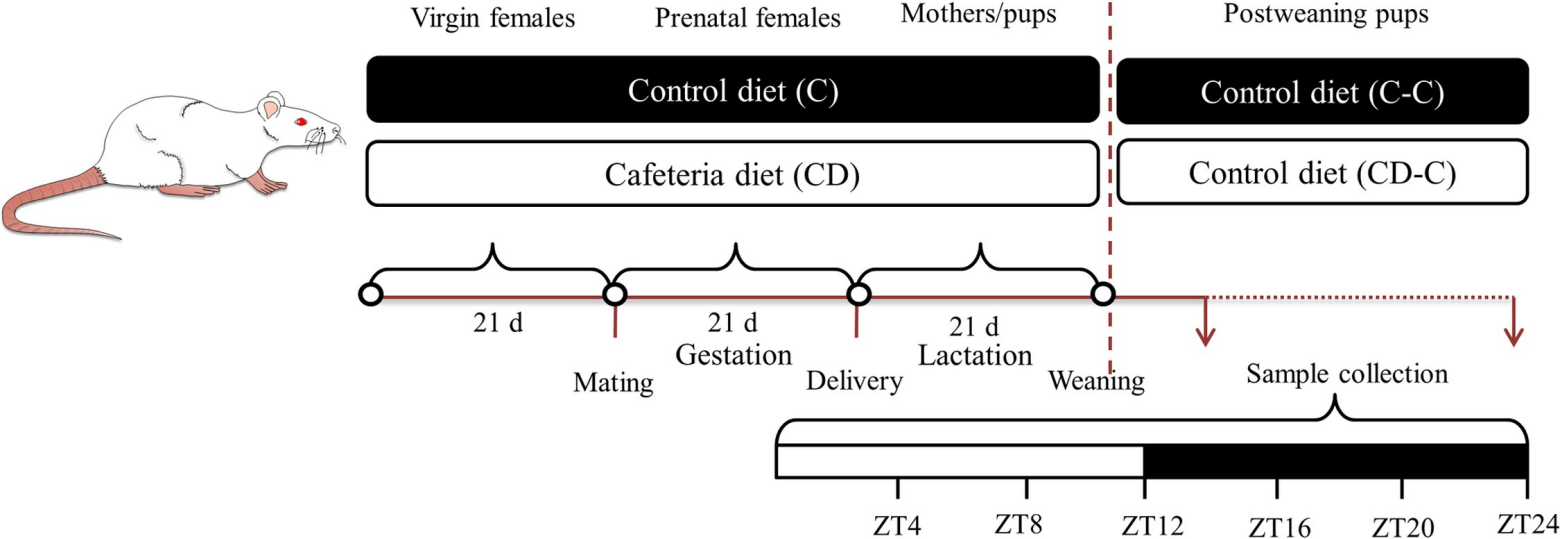
Experimental design. Rat dams were fed either a cafeteria or standard lab rodent diet for 21 days before mating, the whole mating and pregnancy periods, and 21 days of lactation. At weaning, offspring were separated by sex, and those born from dams were malnourished with a cafeteria diet, and those from control dams were fed a standard lab rodent diet. Different tests were performed in the offspring at 40 and 90 days of age: Plasma glucose and triglycerides were determined at six different Zeitgeber Times (Z.T.), and plasma insulin and glucose tolerance tests at ZT4 and ZT20.

The cafeteria diet was based on Purina lab Chow 5008 but supplemented with highly palatable human food: condensed milk, loaf bread, chocolate, cookies, dried coconut, boiled potatoes, and vegetable shortening in equal proportions. All components were mixed and compacted to form pellets with the same ratio of nutrients. The proximal chemical composition of diets is presented in Table 1. Cafeteria diet was administered *ad libitum* for three weeks before and all through mating, gestation, and lactation periods (Fig 1).

**Table 1:** Diet nutrient composition and energy content.

	Diets		
Contents	Control diet (%) 5008	Cafeteria Diet (%)	Control diet (%) 5001
Carbohydrates	50.3	67.16	48.1
Protein	23.6	8.78	23
Fat	6.7	13.73	5.1
Ash	6.1	2.58	7.2
Fiber (crude)	3.3	0.65	5.1
Humidity	10	7.1	11.5
Total Kcal/g	4.15	6.32	2.86

### 2.3. Food intake and morphometric parameters

Food intake of dams and the offspring were recorded every other day until the end of the protocol. The consumption per animal was calculated using the following formula:

(Food placed-food consumed)/number of subjects in the cage. Morphometric parameters: Body weight and length were measured once a week from birth through the end of the protocol. Thoracic and abdominal circumferences were measured at age 21 (i.e., at weaning), 40, and 90 days. Another parameter recorded was the Lee index (as a measure of obesity), equivalent to the human BMI (body mass index) for rats. It divides the cube root of the body weight by naso-anal length multiplied by 1000 [24–27].

### 2.4. Circadian sampling and plasma triglycerides and glucose determination

At 40 and 90 days of age, triglycerides and plasma glucose were sampled at six temporal points (ZT4, ZT8, ZT12, ZT16, ZT20, ZT24; n = 10 per group). In addition, the same animal was taken to 6 blood samples as follows: the blood sample was taken every 24 h plus 4 h of fasting for each time point until completing 6 Z.T.s during a maximum of 7 consecutive days to allow the animal to recover after each sampling.

Heparinized capillary tubes were used to collect blood from the caudal vein to rats anesthetized with Isoflurane (Sofloran Vet®). Glucose and triglycerides in plasma were determined using Spinreact Glucose-LQ and Spinreact Tryglycerides-LQ Kits.

COSINOR adjustment was performed afterward to determine the circadian rhythm parameters for glucose and triglycerides. The mathematical adjustment named COSINOR fits a cosine wave to the raw data and permits to determine: period, acrophase (the time at which the peak of a rhythm occurs), mesor (circadian rhythm-adjusted mean based on the parameters of a cosine function fitted to the raw data) [28] and amplitude (the difference between the maximum and minimum observed values) (CHRONOSFIT® software).

### 2.5. Plasma insulin measurement

Plasma insulin was sampled at two-time points (ZT4, photophase-light-, and ZT20 scotophase-dark-). Rats were fasted 4 hours before blood collection. Insulin concentrations were measured by an ELISA kit (Millipore Cat. # EZRMI-13K). Group n was as follows: C = 5 females/6 males and CD: = 6 females/5 males. The homeostasis model assessment-insulin resistance (HOMA-IR) index was calculated from fasting plasma glucose and insulin concentrations using the following equation: HOMA-IR = [μmol insulin /ml x μmol glucose/ml]/22.5 [29].

### 2.6. Intraperitoneal glucose tolerance test (IpGTT)

At 45 and 95 days of age, after 4–4:30 h of fasting, a glucose tolerance test was carried out at two time points (ZT4 and ZT20). A 10% glucose solution was injected i.p (2g/Kg). An incision was inflicted at the tip of the tail to obtain a drop of blood. Glucose concentration was measured before the glucose injection (time 0) and at 30, 60, 90, and 120 minutes afterward. The same animals were used in both ages and Z.T.s. Group n was as follows: C = 5 females/6 males and CD: = 6 females/5 males. An Accu-Check Performa glucometer measured blood glucose concentration.

### 2.7. Statistical analysis

Data are presented as mean ± S. E. The morphometric parameters, plasma triglyceride and glucose concentrations, IpGTT, HOMA-IR, and area under curves (AUC) were plotted and calculated using GraphPad Prism6. Repeated measures, factorial or one-way ANOVA or Student-t, were used when appropriate, with a Newman-Keuls *post-hoc* test to determine significant differences (P < 0.05) between specific groups and Z.T.’s using the software STATISTICA 12 (Statsoft).

For all procedures, time is expressed as Zeitgeber time (Z.T.), with ZT0 defined as the time when lights were turned on and ZT12 turned off in the light-dark (L.D.) cycle.

All procedures described were in accordance with the ethical guidelines of the Declaration of Helsinki and the National Institutes of Health Guide for the Care and Use of Laboratory Animals (NIH Publications No.8023), Institutional guidelines, and the Government agency SEMARNAT (General Law of Health for Research Studies) (NOM-Z00-2000). The Committee for Academic Ethics and Scientific Responsibility (CEARC) of the Facultad de Ciencias de la UNAM approved all the listed procedures (protocol PI_2021_02_01Bustamante).

## 3. Results

### 3.1 Intrauterine and perinatal cafeteria diet malnutrition causes lower morphometric parameters in the offspring

**Fig 2:**
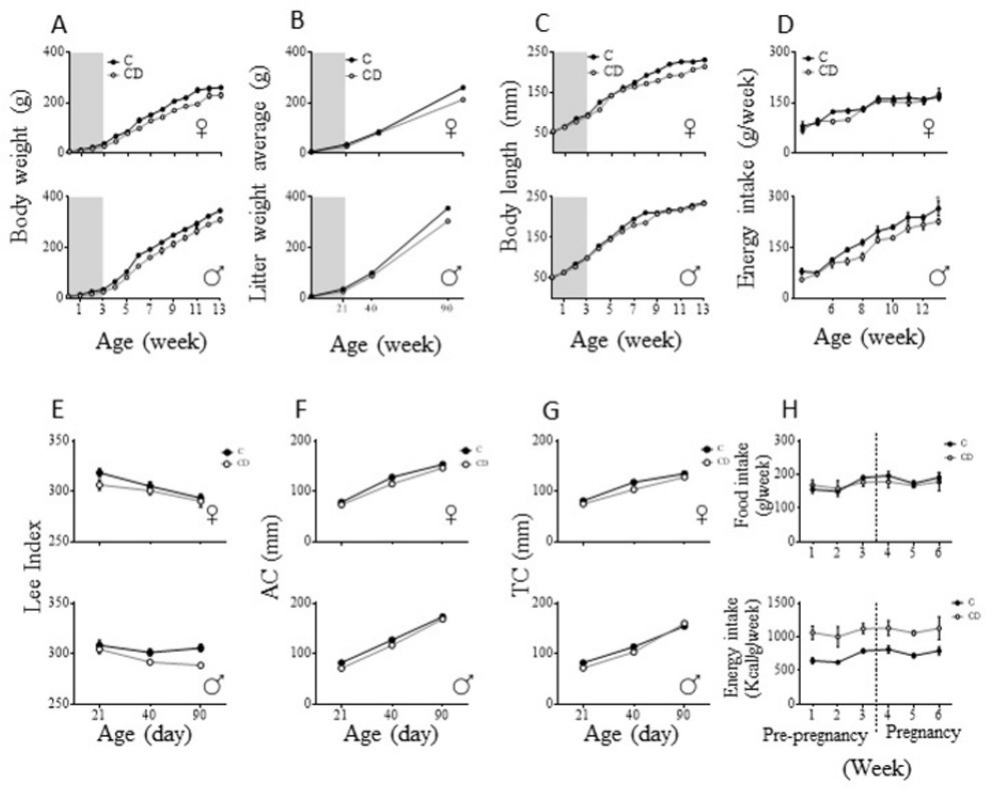
Morphometric developmental parameters of malnourished dams offspring. Accumulative body weight gain (A), average weight increase by litter (B), Body length (C) energetic consumption (D), Lee index (E) abdominal circumferences were measured weekly for 13 weeks (F), as well as thoracic circumference (G). The shaded background corresponds to the lactation period. Food intake (C) was quantified weekly after weaning for offspprings. Food intake and Energy intake for mothers (H). Female C: n = 10, female CD: n = 10, male C: n = 12, male CD: n = 12. Data are expressed as mean ± SEM.

Dams were exposed to the cafeteria diet three weeks before mating and during pregnancy; at these periods, their food consumption did not differ from control (F_1,8_ = 0.25, *p* = 0.63), however, this means that those in cafeteria diet had more caloric intake (F_1,8_ = 57.46, p < 0.001) (Fig 2H). After birth, for 13 weeks, morphometric parameters of the offspring were constantly measured. Both male and female offspring from CD dams showed lower weight growth rates than controls (Females: F _13, 234_ = 4.13, p < 0.0001; males F_13, 286_ = 2.48, p < 0.0001) (Fig 2A). The changes in body weight development between C and CD offspring is not explained by between litter variation, the average weight of the litters follows the same pattern of growing as weights considered individually (Female litters average F_3, 12_ = 80.83, p < 0.0001; male litters average: F_3, 12_ = 27.20, p < 0.0001) (Fig 2B). Body lengthening of CD offspring showed a lower growth rate in both females (F_13_, _252_ = 6.26_,_ p < 0.0001) and males (F_13_, _308_ = 4.4_,_ p < 0.0001); nevertheless, CD males matched body length of C at week 9 (Fig 2C). The overall food intake showed a reduction in both CD males and females (Females: F_1_, _96_ = 4.47_,_ p = 0.037; males: F_1_, _93_ = 29.73_,_ p < 0.0001) (Fig 2D). Interestingly, the Lee index was different between CD females and males, while CD females showed the same Lee index as C females (F_1,18_ = 3.29, p = 0.08), it was lower in CD males after weaning (40 and 90 days) (F_1,22_ = 11.15, p = 0.003) (Fig 2E). Accordingly, both males and females CD offspring showed shorter both abdominal (females F_1_, _18_ = 8.63_,_ p = 0.009; males F_1_, _18_ = 17.11_,_ p = 0.0006) (Fig 2E) and thoracic circumferences (females F_1_, _18_ = 18.5_,_ p = 0.0004; males F_1_, _18_ = 7.72_,_ p = 0.01) (Fig 2F).

### 3.2 Plasma glucose and triglycerides showed altered circadian patterns

**Fig 3:**
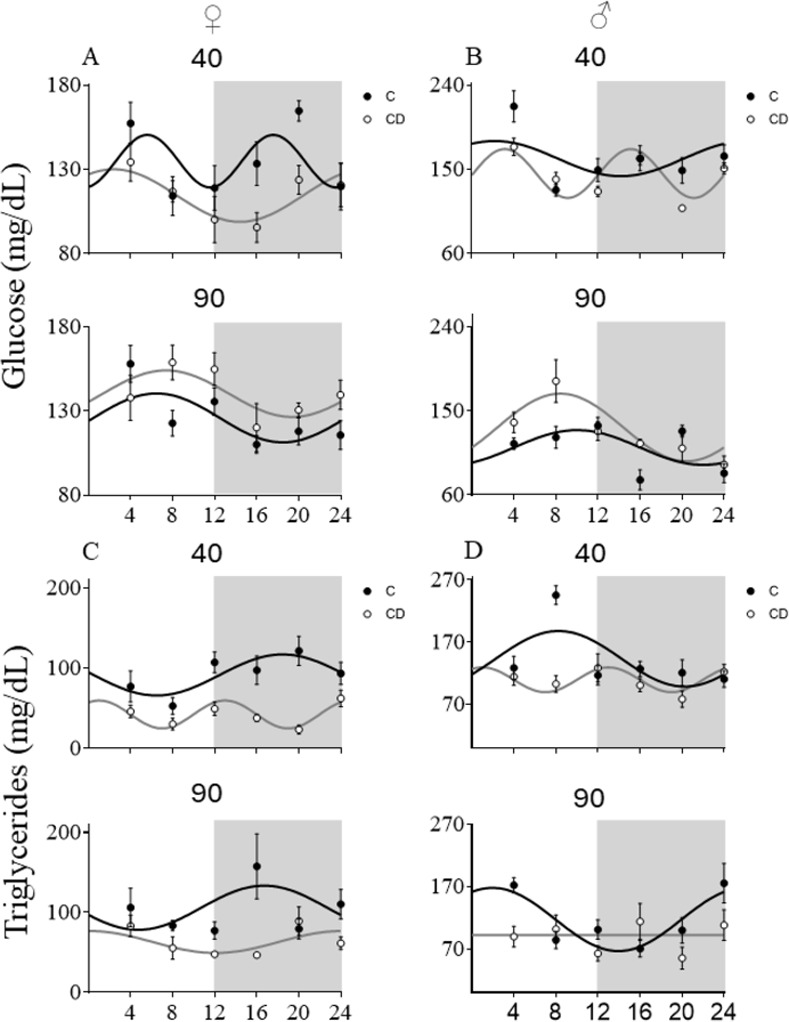
Changes in the circadian rhythmicity of energetic metabolites in the offspring of malnourished dams. Plasma glucose and triglycerides were measured in plasma at six Zeitgeber points, and rhythm was adjusted by the cosinor method. (A) Glucose in 40 and 90 days old females. (B) Glucose in 40 and 90 days old males. (C) Triglycerides in 40 and 90 days old females. (D) Triglycerides in 40 and 90 days old males. C, control diet. CD, cafeteria diet. Shaded background indicates the dark period. ZT0 is defined as the onset of the light period, and ZT12 is defined as the onset of the dark period. n = 10 per group. Data are expressed as mean ± SEM. N-K post-hoc tests: * p < 0.05, ** p < 0.01, *** p < 0.001.

Plasma levels of glucose and triglycerides were quantified at 4-hour intervals throughout 24 h to determine the circadian oscillation pattern. The plasma levels for both energetic metabolites differed between C and CD groups at their temporal distribution. The Cosinor analysis showed that the periodogram for C females at 40 days of age displayed two glucose periods of 12 h, and for CD females, one of 24 h. During the scotophase, the dark period, CD females had lower plasma glucose concentration at ZT16 and ZT20 (F_1, 126_ = 7.51, p < 0.006), a three h of acrophase advance (ZT 3), and lower mesor. At 90 days of age, mesor for CD females was higher than C; at ZT8, CD displayed higher glucose levels (F_1, 122_ = 9.07, p < 0.003; Fig 3A), but the amplitude was similar, although a one h delay in acrophase (C acrophase at ZT 6; Table 2).

**Table 2:** Period, mesor, amplitude, and acrophase from Cosinor analyses of glucose and triglycerides plasm.

*Parameter*	*Age (pnd)*		*Diet*	*Period (h)*	*Amp (mg/dL)*	*Acrophase (Z*.*T*.*)*	*Mesor (mg/dL)*	*F*	*p*
	40		C	12	15.6	5.5	134.7	5.22	= 0.006
		Females	CD	24	15.6	2.4	114.3	7.26	< 0.001
	90		C	24	14.8	6.4	124.2	9.18	= 0.0001
Glucose			CD	24	12.4	7.4	139.6	4.87	< 0.009
	40		C	24	18.7	2.1	160.4	6.30	= 0.002
		Males	CD	12	25.9	3.1	144.6	13.73	< 0.0001
	90		C	24	17.4	10	107.4	4.01	< 0.002
			CD	24	33.7	8.4	127.5	10.16	< 0.0001
	40		C	24	25.7	18.4	91.3	8.27	< 0.001
		Females	CD	12	17.4	1	41.9	16.91	< 0.001
	90		C	24	27.6	16.7	105.8	5.27	= 0.006
Triglycerides			CD	24	13.7	0	62.9	4.68	= 0.01
	40		C	24	41	7.8	143.3	10.21	< 0.001
		Males	CD	12	19.5	1	107.5	3.41	= 0.03
	90		C	24	50.8	1.9	117.1	10.46	< 0.001
			CD	-	-	-	91.9	3.14	> 0.05

C: control diet; CD: cafeteria diet; pnd: postnatal day.

The Cosinor analysis n = 10 per group.

The circadian glucose rhythm of CD males at 40d showed a biphasic behavior, whereas the C rhythm was monophasic. CD 40d males were hypoglycemic versus controls at ZT4 and ZT20 (F_1, 124_ = 10.68, p < 0.001), and the acrophase was delayed for one hour. Both groups of 90d males showed a period of 24 h, but CD males mesor was higher than C (F_1, 108_ = 7.34, p < 0.007), and the acrophase advanced 1.6 hours (Fig 3B and Table 2).

Triglycerides circadian rhythm was biphasic in 40d CD females, while C displayed a monophasic rhythm; the CD group had lower plasma triglycerides in the scotophase, where the low concentration point (batiphase) was (F_1, 126_ = 11.23, p < 0.001), an acrophase delay of 6 hours at Zt1 in comparison to C acrophase at ZT18. In addition, the mesor was lower in CD than in C. Both female groups at 90d showed a period of 24 h, with an acrophase delayed seven h (ZT0) in C.D.s. CD females also had a lower level (mesor) of triglycerides in plasma during scotophase ZT16 (F_1, 126_ = 19.34, p < 0.001) (Fig 3C and Table 2). CD males of 40d also showed a lower mesor, an advance in acrophase of 6 hours (ZT1), and lower levels of triglycerides during photophase ZT 8 (F_1, 126_ = 11.23, p < 0.001) (Fig 3D and Table 2). At 90d, triglycerides for CD males did not show circadian oscillation, but the concentration was lower than C males at ZT4 and 24 (F_1, 124_ = 7.37, p < 0.007) (Fig 3D and Table 2).

### 3.3 Glucose tolerance test at ZT4 (light) and ZT20 (dark)

**Fig 4:**
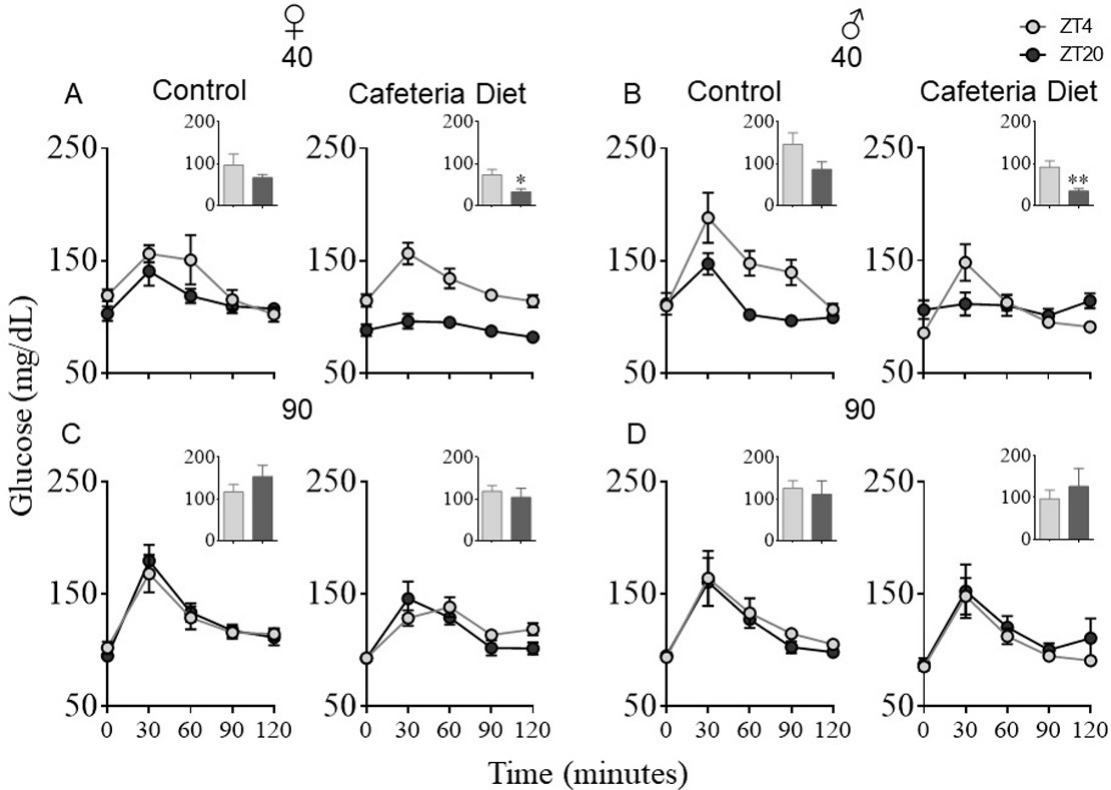
Offspring of malnourished dams presented higher tolerance to glucose at scotophase IpGTT was performed at a light time (ZT4) and a nighttime (ZT20). (A) 40 days old females; (B) 40 days old males; (C) 90 days old females; (D) 90 days old males. AUC area under the curve in arbitrary units. Females C: n = 5, Females CD: n = 6, Males C: n = 6, Females CD n = 5. Data expressed as mean ± SEM. t-tests * p < 0.05, ** p < 0.001.

In order to elucidate changes in glucose handling capacity during both phases of the photoperiod, glucose tolerance tests were performed at a light time (ZT4) and a dark time (ZT20), as previously reported [30]. At 40d CD offspring, both females (Fig 4A) and males (Fig 4B) showed a significantly faster blood glucose decrease during darkness (ZT20) than at light time (ZT4) with significantly lower areas under the curve (AUC) (Females: t_9_ = 2.6, p = 0.03; males: t_9_ = 3.8, p = 0.004). C 40d groups exhibited a similar tendency, but with higher peaks at 30 min and an AUC not significantly different (Females: t_8_ = 0.1.08, p = 0.30; males: t_9_ = 0.1.7, p = 0.11). At a later stage of development (90 days old), glucose tolerance differences between offspring of cafeteria diet dams and those of control ones vanished in both sexes (Fig 4C and 4D). C and CD groups showed no differences in glucose peaks nor AUC between light and dark times for any C and CD group.

### 3.4 Plasma insulin and insulin resistance at ZT4 and ZT20

**Fig 5:**
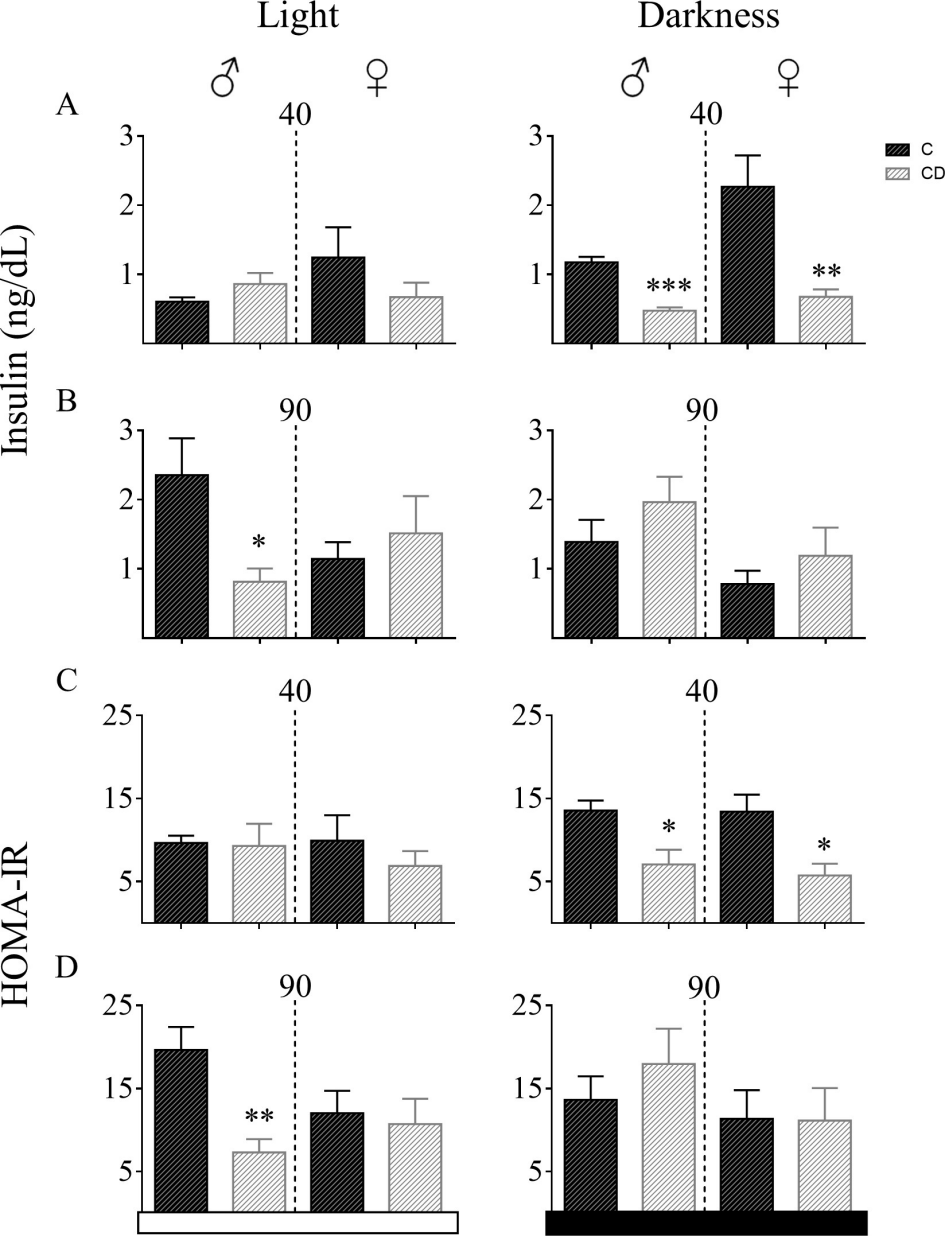
Insulin release and HOMA-IR index at different developmental stages and Zeitgeber times. Insulin and glucose were measured at ZT4 and ZT20 in CD and C groups in both sexes at 40 and 90 days of age. (A) Insulin plasma concentrations. (B) HOMA-IR. Females Control (C): n = 5, females from Cafeteria Diet (CD) dams: n = 6, males C: n = 6, females CD n = 5. Data expressed as mean ± SEM. t-tests * p < 0.05, ** p < 0.01, *** p < 0.001.

During scotophase (ZT20), offspring of malnourished dams showed a glucose tolerance curve with low blood glucose concentrations during the time monitored. To address the origin of this effect, at Z.T.s 4 and 20, both insulin circulating levels were measured, and calculated the HOMA index. The HOMA represents insulin resistance with the relationship between fasting glucose and insulin concentrations [8]. During ZT20, insulin levels in males and females of 40d old CD were lower than in C groups (females: t_10_ = 3.4, p = 0.007; males: t_10_ = 7.6, p < 0.0001). However, this effect does not appear during daytime (females: t_8_ = 1.18, p = 0.27; males: t_10_ = 0.1.48, p = 0.17; Fig 5A). At 90 days, only in males and during time ZT4, CD group showed lower insulin (t_9_ = 2.5, p = 0.03). In females this difference was not found (t_10_ = 0.6, p = 0.54) neither during dark-time (ZT20) in both sexes (males: t_9_ = 0.78, p = 0.45, females: t_10_ = 0.9, p = 0.38; Fig 5A).

Previous studies suggested that low protein consumption leads to a decrease in insulin resistance^19^. Therefore, the HOMA-IR index was calculated to confirm if low protein content in the cafeteria diet increases insulin sensitivity, causing a high glucose tolerance and low insulin levels observed at ZT20 in 40-day old rats. Both male and female CD rats showed similar levels of insulin resistance (HOMA-IR) as their C counterparts at ZT4 (females: t_8_ = 0.86, p = 0.41; males: t_10_ = 0.13, p = 0.90). However, during ZT20, both male and female CD rats showed lower HOMA-IR than C rats (Females: t_8_ = 3.076, p = 0.015; males: t_10_ = 3, p = 0.01). At 90 days of age, only males and ZT4 exhibited a difference versus C males (t_8_ = 3.8, p = 0.005; females: t_10_ = 0.32, p = 0.75). At ZT20, both CD and C rats showed similar insulin resistance index in both sexes (males: t_10_ = 0.84, p = 0.41; females: t_10_ = 0.04, p = 0.96) (Fig 5B).

### 4. Discussion

The present results show that exposure to an industrialized, highly palatable, and poorly nutritious (high fat and low protein content) diet in dams during pregnancy and lactation modifies the circadian oscillation pattern of energetic metabolites. The present data showed a modification in the circulating concentrations and rhythmicity of triglycerides and glucose in juvenile and adult offspring of dams exposed to a cafeteria diet. The main rhythmicity changes were in acrophase and amplitude. These changes occur with a higher tolerance to glucose and an increased sensitivity to insulin in both sexes. However, these changes in glucose and insulin do not persist in the adult stages. Additionally, the present results show that a perinatal cafeteria diet, high in caloric content and low in protein, delays the growth of the offspring in morphometric parameters such as weight and length in female and male animals.

Maternal malnutrition modifies the glucose metabolism in the offspring, generating changes in the basal concentrations during life and circadian rhythmicity. These modifications generated during development can be expressed differentially during different life stages but are consequences of programming during early development that likely cannot be reverted with a balanced diet during juvenile or adult stages. In the present study, we used PND-40 and PND-90 because these ages allowed us to compare two stages of life, juvenile organisms (but metabolically mature) and young adults, showing that the effects generated by maternal malnutrition impact young or adult offspring in the same way. These findings are consistent with reports that malnutrition during gestation and lactation affects juvenile and young adult offspring between 30 (4 weeks) and 56 days (8 weeks), impacting feed intake, lipid metabolism, glucose metabolism, and the rhythmic expression of clock genes [2, 31].

Furthermore, the present study shows that the rhythmicity of energetic metabolism is also altered during the day, which can trigger other pathologies in other physiological systems. The molecular clock of the peripheral oscillators, e.g., liver, pancreas, skeletal muscle, and adipose tissue, regulate the energetic homeostasis rhythmicity [32]. The expression of Bmal and Clock, positive factors in the molecular clock loop, is linked to the circadian expression of genes that participate in liver lipogenesis [17], gluconeogenesis, and the daily rhythmicity of glucose and triglycerides [33]. Conversely, the response to fasting in the liver relates to the negative components of the loop (Per and Cry), which promote the storage of glucose in the form of glycogen [34]. Importantly, the loss of synchronicity of the clock genes in the peripheral oscillators implicates an imbalance in lipid and carbohydrate metabolism.

Furthermore, alterations in the rhythmicity of clock genes in mice show attenuation in the rhythmic patterns and concentration decrease of insulin, glucose, and triglycerides [20, 35]. All these data together demonstrate that the integrity of the molecular clock components is necessary for regulating and synchronicity of the circadian rhythm of energetic metabolism. Therefore, alterations in the molecular clock caused by maternal malnutrition could explain the oscillator modifications observed in the present study.

The common use of highly industrialized food with high-added fat and simple sugars has increased worldwide. Within the groups highly exposed to such diet are women at reproductive stages, with consequences in the early phases of the offspring development, such as programming of their energetic metabolism. Metabolic programming is fundamental for efficient interaction with the environment. The present results show that exposure to a cafeteria diet during early development generates repercussions for life in the offspring, even after long periods of exposure to a balanced diet. Then, the offspring of malnourished dams are permanently affected in their life quality.

Deficient diets during gestation can generate modifications in the offspring. Previous literature showed that malnourished mice during gestation with low content of protein in their diet present low weight during the first weeks of life, and after a catch-up, they show obesity and insulin resistance in their adult life [32]. However, the present results show that at 13 weeks, rats do not show obesity and improve their insulin sensitivity. As shown in previous studies, more time would be necessary to see if this effect is maintained or inverted with the aging process. Several studies in rodents showed that unbalanced nutrition in early development stages, e.g., gestation or lactation, yields metabolic impairments, such as hyperglycemia and insulin resistance [36, 37]. Nevertheless, most of these studies only evaluate changes in adult stages, while the present study focuses on the whole maturation process evaluating the first 13 weeks of age, which could explain a possible discordance. Furthermore, few studies have evaluated metabolic changes presented in the offspring during several life stages; most studies only evaluate the changes in single stages where metabolic changes are present, such as the increase in glucose concentrations [6, 38], modifications of insulin sensitivity [6, 39, 40] and alterations in lipid metabolism [41–43].

Cafeteria diets are effective in increasing metabolic alterations that allow the development of metabolic syndrome [37]; those alterations depend on the life stage of administration. Pups exposed during gestation and lactation to a high-fat diet (H.F.) show that despite the similar weight at birth, at six months, they have more adipose tissue and higher triglycerides [24]; accordingly, in posterior stages, the offspring of malnourished rats can show a tendency to obesity during the aging process. Conversely, higher insulin sensitivity, lower weight gain, and high circulating triglycerides result from low-protein diets. The fatty acid esters of hydroxy fatty (FAHFAs) have beneficial metabolic effects, including higher insulin sensitivity [44]. The diet can modify the rhythms of metabolites; however, few studies have evaluated the effect of maternal malnutrition on the circadian rhythms of energetic metabolites of the offspring. In this context, a previous study showed that a low-protein diet at gestation modifies the master oscillator and the peripheral oscillators, generating a predisposition for adiposity and weight gain in the adult stages [45].

Adamovich, Rousso-Noori [14] showed that total triglycerides present a circadian oscillation serving as indicators of the proper functioning of energy metabolism in animal and human models. The decrease in triglycerides and lower body weight seem to be the reason for higher insulin sensitivity in both females and males at the juvenile stage in the present study. Other commonly evaluated lipid is the cholesterol which is also regulated by circadian rythimicity, however, we focused in triglycerides as a main energy source interconnected with insulin a glucose metabolism, The peripheral oscillators have a fundamental role in metabolism, particularly the liver, the adipose tissue, and the pancreas. Those tissues are involved in metabolic homeostasis and its temporal regulation. However, the dynamics of that regulation can be altered by dysregulation of the phase relationship between the different peripheral oscillators and the central SCN as an effect of exposure to a diet elevated in fat [46]. The molecular machinery known as clock genes sustains the rhythmicity of oscillators. This molecular machinery adjusts to the rhythmicity of the different peripherical and central oscillators; it also regulates the energetic metabolism, including glucose levels and triglycerides. Specifically, the genes Per2 regulate nuclear receptors that participate in adipogenesis and Insulin sensitivity [47].

The intrauterine and perinatal exposure to a cafeteria diet affects the programming of the energy metabolism via modification at critical developmental periods producing mid and long-term alterations, not only in the peripheral but also in the central oscillator. The high fat/low protein diet can lead to severe maladjustment in the glucose and triglycerides rhythm, unfolding severe metabolic modifications that can generate metabolic syndrome. An increase in visceral adipose tissue, hyperinsulinemia, and glucose intolerance [37] are symptoms of metabolic syndrome. There is no consensual definition of a standard cafeteria diet composition; though a low protein/high carbohydrate is the typical cafeteria diet used in experimental approaches, this can generate different and divergent results compared to previous studies. The cafeteria diet is characterized by both low-protein and high-fat content; however, these two components by separate have distinct effects. Both diets impact the rhythmicity of energetic metabolites; however, the present results more closely resembles a protein-low diet, resulting in a lower weight and similar glucose tolerance curves in adults [20]. Contrarily, the high-fat diet is obesogenic, which is inconsistent with the data of the present study [48]. Nutrition is crucial in programming physiological responses, leading to significant alterations in mothers and offspring. Research has highlighted sexual dimorphism in various metabolic parameters, particularly in glucose regulation. For instance, insulin signaling (IRS1), which is higher in males, and glucose-induced expression of GLUT4 in early stages affect glucose regulation differently between males and females [49]. This sexual dimorphism extends to metabolic impacts, with males showing increased insulin resistance. Moreover, the effects of nutrition on adiposity vary with age, with females displaying a greater predisposition to adiposity as they age. Additionally, adult males exhibit higher concentrations of triglycerides due to decreased signaling of hepatic lipids and insulin [50]. These findings underscore the intricate interplay between nutrition, sex, and metabolic outcomes, highlighting the need for further research. In view of the results, the next step is to analyze the expression of clock genes in both the peripheral oscillators and the suprachiasmatic nucleus, to estimate the modulation in the molecular clock caused by perinatal malnutrition and their influence in energetic metabolites.

In conclusion, the exposure of dams to a cafeteria diet during gestation and lactation programs the energy metabolism of the offspring, generating phase changes in the circadian rhythmicity of glucose and triglycerides. Thus, alterations in rhythmicity include hyperglycemia in a resting stage and increased insulin sensitivity in the activity phase in males and females. The metabolic maladjustments can predispose the offspring to generate metabolic syndrome in later stages of life. Due to a cafeteria diet in the early stages of development, malnutrition programs the energetic metabolism of the offspring, modifying the rhythmicity of plasma concentrations of glucose and triglycerides in juvenile stages that prevail for life.
